# Thermal ablation versus liver resection for hepatocellular carcinoma in patients with cirrhosis: a systematic review and meta-analysis of propensity-score matched studies

**DOI:** 10.1007/s10238-023-01285-w

**Published:** 2024-02-01

**Authors:** Qiuxia Wei, Shiyu Xiong, Wanrong Luo, Ming Liang, Baoming Luo

**Affiliations:** Department of Ultrasound, Sun Yat-sen Memorial Hospital, Sun Yat-sen University, 107 West Yanjiang Road, Guangzhou, 510120 China

**Keywords:** Hepatocellular carcinoma, Thermal ablation, Liver resection, Outcome

## Abstract

The outcomes of cirrhotic patients with hepatocellular carcinoma (HCC) after thermal ablation (TA) versus liver resection (LR) are debated. We aimed to compare the overall survival (OS), disease-free survival (DFS), and operative outcomes after TA and LR for HCC in patients with cirrhosis. Until November 15, 2022, we searched PubMed, Embase, and Cochrane databases by using Medical Subject Heading terms and other terms, and used the Newcastle-Ottawa literature evaluation scale to assess the quality of selected studies. OS, DFS, and operative outcomes were extracted and analyzed. The meta-analysis showed that 5 propensity-score matched (PSM) studies including 933 patients (463 TA vs. 470 LR) were included. After analysis, TA and LR had similar results at 1-year OS (odds ratio [OR] 1.68; 95% confidence interval [CI] 1.01–2.78; *P* = 0.05) and 3-year OS (OR 0.76; 95% CI 0.56–1.04; *P* = 0.08), whereas LR increased 5-years OS (OR 0.37; 95% CI 0.18–0.74; *P* = 0.005). In addition to the DFS, the 1-year DFS was significantly higher in patients with LR. However, there were no obvious differences in 3-year and 5-year DFS when comparing TA and LR. The length of operative time and hospital stay were longer in the LR group. Besides, the LR group had significantly higher rate of perioperative blood transfusions and major complications. Our research proved that LR took advantage of OS and DFS for HCC patients with cirrhosis. Additional well-designed randomized controlled trials are needed.

**Supplementary Information:**

The online version contains supplementary material available at 10.1007/s10238-023-01285-w.

## Introduction

Liver cancer is the sixth most common cancer worldwide, and the third leading cause of death worldwide killing more than 800,000 people each year [1]. The most widely recognized condition that predisposes HCC is hepatic cirrhosis, and almost 90% of HCC patients have chronic infections with the hepatitis B or C virus [2]. Its incidence is expected to rise and peak within the next 20–30 years mirroring the predicted worldwide epidemiology of viral hepatitis.

Patients with cirrhosis tend to have stiff, fibrotic liver parenchyma, elevated portal pressures, thrombocytopenia, and impaired clotting which adds further complexity to the choice of HCC treatment. There are many therapies for HCC, including surgical resection, liver transplantation, radiofrequency ablation, microwave ablation, transcatheter arterial chemoembolization, targeted therapy, and immunotherapy. Recently, a meta-analysis including seven RCTs and 18 matched nonrandomized trials had shown that LR was better than radiofrequency ablation (RFA) when considering the recurrence-free survival and incidence of local recurrence but these two therapies made the same effect on OS between LR and RFA [3]. That is consistent with the international guideline for the management of HCC, suggesting that RFA and LR as the standard treatments for HCC patients with single HCC < 2 cm or single or 2–3 nodules < 3 cm with preserved liver function [4]. An RCT comparing the efficacy of microwave ablation (MWA) and RFA for the treatment of HCC patients reported that MWA was at least as safe and effective as RFA [5]. Furthermore, a meta-analysis of four RCTs and ten cohort studies showed that MWA and RFA had the same effect on complete ablation, local recurrence, DFS, OS, and the major complication rate [6].

However, the choice of LR or TA for patients with cirrhosis is still controversial. Several retrospective studies showed that patients with LR had better OS and DFS than those with TA [7–9]. Whereas, Johannes et al. [10] found no difference in OS between these two treatments. On the contrary, Tito et al. proposed that compared with LR, TA is less invasive, has less complication rate and lower cost, and should be the best choice for cirrhotic patients with HCC [11]. A meta-analysis reported no obvious differences in 3-year and 5-year OS and DFS between patients with cirrhosis who underwent TA and those who underwent LR [12]. Since most of the studies included in these analyses were retrospective and many case-matching studies compared LR and TA in patients with HCC have been updated, we conducted a meta-analysis by selecting studies of cirrhotic patients with HCC who had got the TA or LR treatment to evaluate the OS, DFS, and operative outcomes.

## Methods

This research followed the Preferred Reporting Items for Systematic Reviews and Meta-Analyses (PRISMA) statement standards [13] and Assessing the Methodological Quality of Systematic Reviews (AMSTAR) guidelines [14].

### Inclusion and exclusion criteria

#### Inclusion criteria

1. RCTs or PSM studies in English;
2. Reporting outcomes of thermal ablation (RFA or MWA) versus liver resection;
3. Adult patients (≥ 18 years) with confirmed cirrhotic HCC.

#### Exclusion criteria

1. Journal reviews, news, opinions of experts and editors, articles that have no original data, case reports and studies without a control group, data of the articles that were overlapping;
2. Studies that included patients who underwent liver resection for non-HCC pathologies;
3. Reports that included patients without cirrhosis of the liver;
4. Articles not reporting the outcomes of interest.

### Literature search strategy

A combination of Medical Subject Headings (MeSH) and keywords were used to search PubMed, Cochrane databases, and Embase until November 15, 2022. We conducted the search strategy based on the following: (MeSH exploded “thermal ablation” OR key words “radiofrequency ablation” OR “RFA” OR keywords “microwave ablation” OR “MWA”) and (MeSH exploded “Hepatectomy” OR keywords “resection”) and (MeSH exploded “Carcinoma, Hepatocellular” OR key words “hepatocellular carcinoma” AND “HCC”). We registered our protocol with PROSPERO (ID: CRD42022375981).

### Literature quality assessments

The results were cross-validated by two investigators separately assessing the included papers' bias risks by using the Newcastle-Ottawa literature evaluation scale with a maximum score of 9 [15]. High-quality papers are defined as above or equal to 7 points.

### Data extraction

A standardized extraction form was used to extract baseline characteristics and outcomes from the included studies. Key study characteristics were extracted. The numbers of surviving patients for OS and DFS were calculated from the Kaplan–Meier curves. The primary outcomes were 1-year, 3-year, and 5-year OS. Secondary outcomes were 1-year, 3-year, and 5-year DFS. Other outcomes included operative time, hospital stay, the perioperative blood transfusion rate, and major complications rate (including liver failure, cardiovascular complications, infection, pleural effusion, and ascites).

### Statistical analysis

For dichotomous outcomes (OS, DFS, perioperative blood transfusion, and major complications rate), we calculated the odds ratio and corresponding 95% confidence interval. For continuous outcomes (operative time and hospital stay), the mean difference (MD) and corresponding 95% confidence interval were calculated. We used the chi-square-based Q statistical test to evaluate the heterogeneity between studies. Heterogeneity was considered significant with *p* ≤ 0.10, and pooled OR was evaluated via a random-effect model (the Mantel–Haenszel method) [16]. On the contrary, if the statistical study did not show heterogeneity (*p* > 0.10), a fixed effects model (the Mantel–Haenszel method) was performed [17]. Assessing publication bias was done by using a funnel plot. Statistical significance was determined by *P* values with less than 0.05, which were all two-sided. We used RevMan 5.2 software for our meta-analysis.

## Results

### Screening process of literature

**Fig. 1:**
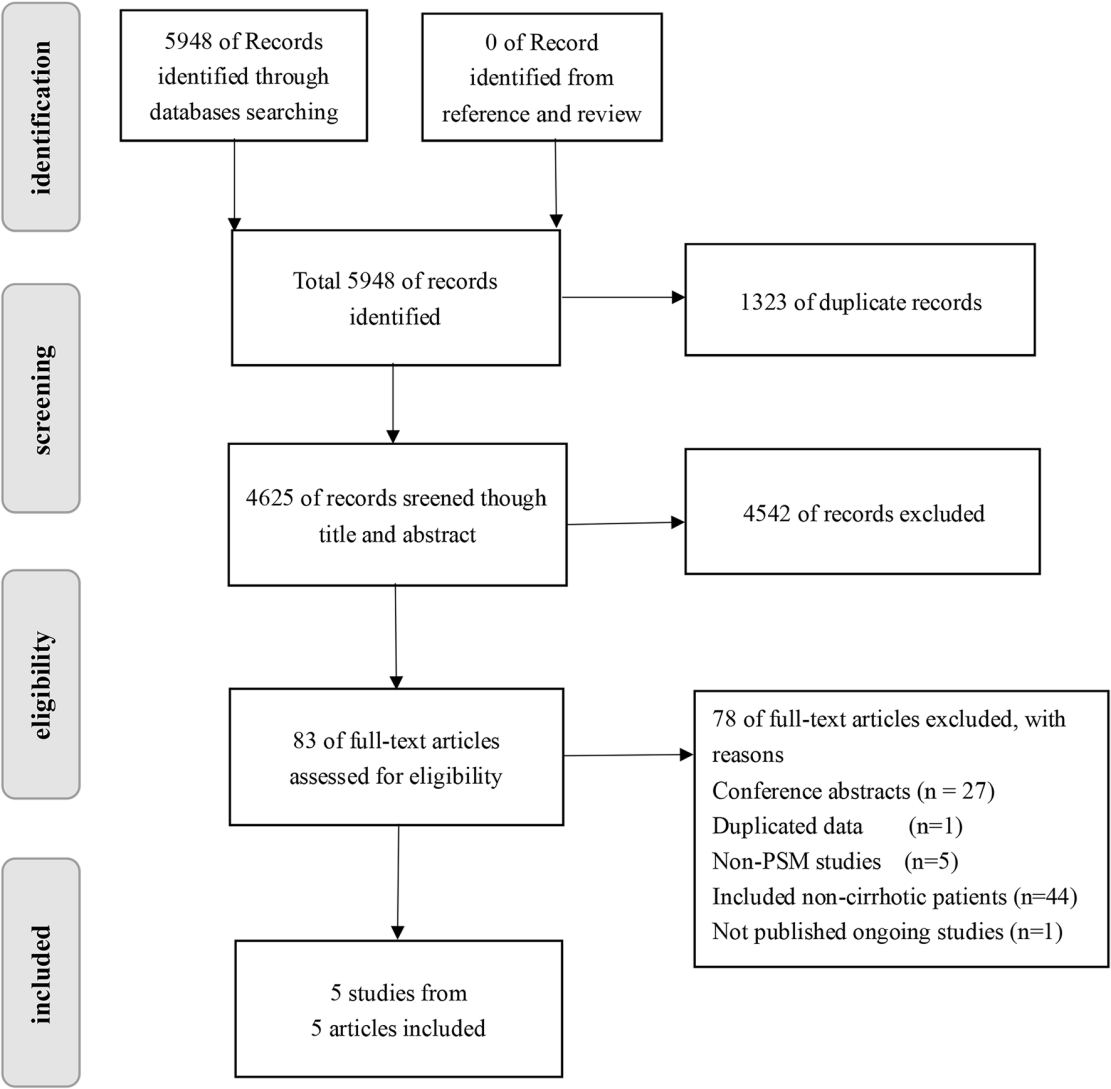
Flow diagram of study selection

Based on the process of searching and selecting illustrated in Fig. 1, 1323 duplicate studies were found out of 5948 records. The titles and abstracts of 4542 papers were deemed uncorrelated to the study's content, and after reading the main text, 78 papers were eliminated. A total of five papers were considered to be applicable (NOS score ≥ 7), containing four retrospective studies and one prospective study [18–22]. According to these five studies, 933 patients underwent PSM, 463 of them (50.6%) underwent TA and 470 of them (50.4%) underwent LR. Table 1 summarized the above information.

**Table 1:** List of included studies

First author	Liver function	Country	Type of study	Tumor size	Tumor number	Total sample	Comparison	LR	TA	NOS score
Santambrogio [18]	Child-Pugh A (84–92%)	Italy France	Prospective study	≤ 2 cm	1	152	LR versus RFA	76	76	8
Delvecchio [19]	Child-Pugh A (81–100%)	Italy France Spain Switzerland	Retrospective study	≤ 3 cm	1	52	LR versus RFA	26	26	7
Pompili [20]	Child-Pugh A (100%)	Italy	Retrospective study	≤ 3 cm	1	232	LR versus RFA	116	116	7
Conticchio [21]	Child-Pugh A (84–85%)	Italy France Spain	Retrospective study	≤ 3 cm	≥ 2	272	LR versus RFA	136	136	7
Lee [22]	Child-Pugh A (100%)	Korean	Retrospective study	≤ 3 cm	≤ 2	225	LR versus RFA	116	109	8

*LR* Liver resection, *TA* Thermal ablation

### Baseline characteristic

Table 1 summarized the characteristics of the included studies. Most of the patients were Child-Pugh class A liver cirrhosis. Of these studies, five were carried out in Italy, three in France, two in Spain, one in Switzerland, and one in Korea. Four studies had comparisons on 1-year OS and 3-year OS and three on 5-year OS. Two studies had comparisons on 1-year DFS, 3-year DFS, and 5-year DFS, the operative time, hospital stay, and the perioperative blood transfusion rate. Four studies had comparisons on major complications rate.

### Meta-analysis results

#### The comparison between TA and LR regarding OS

**Fig. 2:**
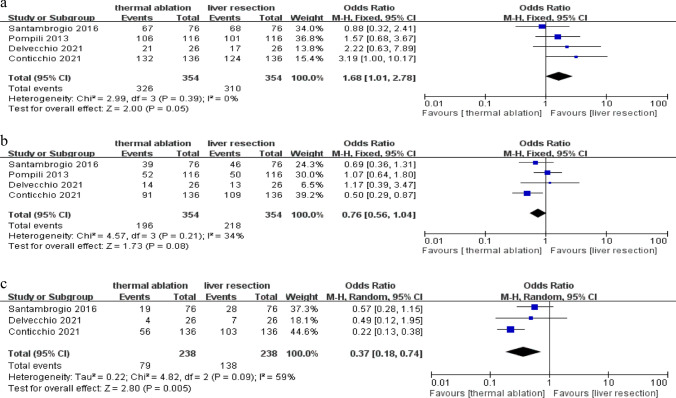
**a** Forest plot of the 1-year OS. **b** Forest plot of the 3-year OS. **c** Forest plot of the 5-year OS

1-year OS incidence comparing TA with LR was compared in four studies including 708 patients. There was no significant heterogeneity among the studies found by heterogeneity testing (*P* = 0.39,* I*^*2*^ = 0%) and nonsignificant publication bias (Fig. S1a). We used a fixed effects model. Figure 2a revealed that the 1-year OS in the TA group (326 of 354 patients) was 92.1% and in the LR group (310 of 354 patients) was 87.6%. Based on the meta-analysis, the pooled OR of 1.68 (95% CI 1.01–2.78; *P* = 0.05) showed that 1-year OS in TA did not differ significantly from LR.

3-year OS incidence comparing TA with LR was compared in four studies including 708 patients. There was no significant heterogeneity among the studies found by heterogeneity testing (*P* = 0.21, *I*^*2*^ = 34%) and nonsignificant publication bias (Fig. S1b). We used a fixed effects model. Figure 2b revealed that the 3-year OS of the TA group (196 of 354 patients) was 55.4% and in the LR group (218 of 354 patients) was 61.6%. Based on the meta-analysis, the pooled OR of 0.76 (95% CI 0.56–1.04; *P* = 0.08) showed that there were no obvious differences in 3-year OS comparing TA with LR.

Figure 2c showed the comparisons of 5-year OS between TA and LR in three studies including 476 patients, and there was heterogeneity among the studies found by heterogeneity testing (*P* = 0.09,* I*^*2*^ = 59%). However, there was no significant publication bias (Fig. S1c). We used a random effects model. The 5-year OS was 33.2% (79 of 238 patients) in the TA and 58.0% (138 of 238 patients) in the LR. A pooled OR of 0.37 (95% CI 0.18–0.74; *P* = 0.005) showed that the 5-year OS of LR was significantly higher than TA, based on the results of the meta-analysis.

#### The comparison between TA and LR regarding DFS

**Fig. 3:**
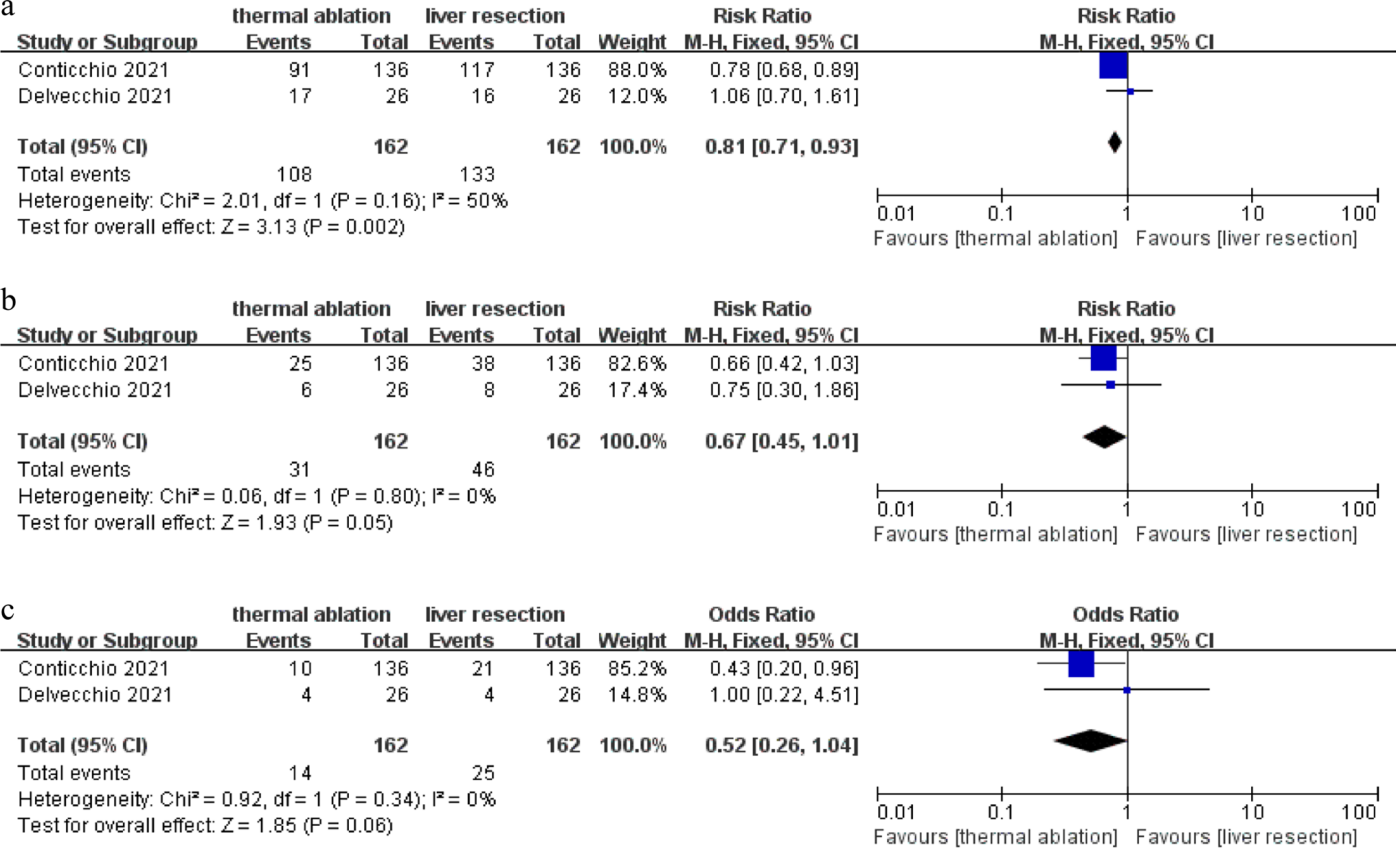
**a** Forest plot of 1-year DFS. **b** Forest plot of 3-year DFS. **c** Forest plot of 5-year DFS

The incidence of 1-year DFS comparing TA with LR was conducted in two studies including 324 patients, and there was no significant heterogeneity among the studies found by heterogeneity testing (*P* = 0.16, *I*^*2*^ = 50%) and there was no evidence of publication bias based on the funnel plot (Fig. S2a). We used a fixed effects model. As shown in Fig. 3a, the 1-year DFS in the TA group was 66.7% (108 of 162) and in the LR group was 82.1% (133 of 162). There was a higher 1-year DFS for the LR group compared to the TA group, based on the findings of the meta-analysis, with a pooled OR of 0.81 (95% CI 0.71–0.93; *P* = 0.002).

The incidence of 3-year DFS between TA and LR was compared in two studies including 324 patients, and there was no significant heterogeneity among the studies found by heterogeneity testing (*P* = 0.80, *I*^*2*^ = 0%) and nonsignificant publication bias (Fig. S2b). We used a fixed effects model. As shown in Fig. 3b, the 3-year DFS in the TA group was 19.1% (31 of 162) and in the LR group was 28.4% (46 of 162). There was no obvious difference in 3-year DFS comparing TA with LR, with a pooled OR of 0.67 (95% CI 0.45–1.01; *P* = 0.05).

The incidence of 5-year DFS between TA and LR was compared in two studies including 324 patients, and there was no significant heterogeneity among the studies found by heterogeneity testing (*P* = 0.34, *I*^*2*^ = 0%) and nonsignificant publication bias (Fig. S2c). We used a fixed effects model. As shown in Fig. 3c, the 5-year DFS in the TA group was 8.6% (14 of 162) and in the LR group was 15.4% (25 of 162). There was no obvious difference in 5-year DFS comparing TA with LR, with a pooled OR of 0.52 (95% CI 0.26–1.04; *P* = 0.06).

#### The comparison between TA and LR regarding operative outcomes

**Fig. 4:**
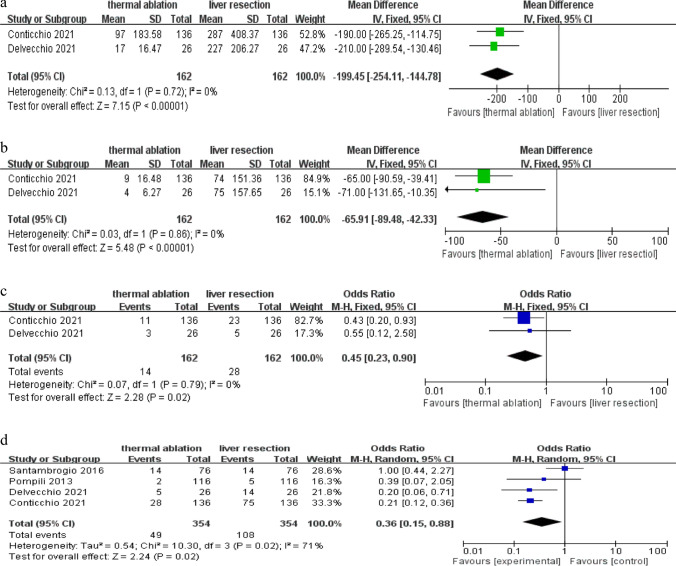
**a** Forest plot of operative time. **b** Forest plot of hospital stay. **c** Forest plot of perioperative blood transfusion rate. **d** Forest plot of major complications rate

As Fig. 4a–c showed, two studies including 162 patients compared the operative time, hospital stay, and the perioperative blood transfusion rate between TA and LR. There was no significant heterogeneity among the studies and nonsignificant publication bias was found in operative time and perioperative blood transfusion rate (Fig. S3a, c). However, there was obvious publication bias based on the funnel plot in the hospital stay (Fig. S3b). We used a fixed effects model. As a result, the length of operative time and the hospital stay of the LR group were significantly longer than that of the TA group (MD = − 199.45, 95% CI − 254.11 to − 144.78, *P* < 0.001 and MD = − 65.91, 95% CI − 89.48 to − 42.33, *P* < 0.001, respectively). There was also an obvious difference in perioperative blood transfusion rate between TA and LR, with a pooled OR of 0.45 (95% CI 0.23–0.90; *P* = 0.02). The rate of major complications between TA and LR was compared in four studies including 354 patients, and there was heterogeneity among the studies found by heterogeneity testing (*P* = 0.02,* I*^*2*^ = 71%) (Fig. 4d). However, there was no significant publication bias (Fig. S3d). We used a random effects model. The result showed that the TA group had a lower major complications rate than the LR group with a pooled OR of 0.36 (95% CI 0.15–0.88; *P* = 0.02).

## Discussion

In this systematic review and meta-analysis of patients with cirrhotic HCC who underwent TA or LR for treatment, the 5-year OS and 1-year DFS were higher in the LR group. But we did not detect any obvious difference in 1-year and 3-year OS and 3-year and 5-year DFS between TA and LR groups. The finding were in line with our previous meta-analyses which also showed better survival at 5 years in the LR group [23].

It is not the first meta-analysis to compare TA and LR in HCC patients when considering cirrhosis background. A meta-analysis published recently including 11 observational studies from Asia, Europe, and North America, compared RFA with LR for the treatment effect of cirrhotic HCC patients [12]. Their results found that there was no difference in OS and DFS when studies were limited in HCC patients with cirrhosis who were equally eligible for LR and RFA or limited to those with only solitary tumors < 3 cm. However, these results were not completely consistent with our meta-analysis findings. First, using PSM can reduce bias and offer homogeneity between baseline data from different groups [24]. Considering there were no RCTs found, we included PSM studies for matching rationally two groups to accomplish results similar to those in RCTs. Second, studies comparing LR and TA in patients with cirrhotic HCC had been updated. Our research yielded refreshed results by including the 3 newly published articles. Third, we could get more general conclusions without limiting the grade of liver function when including the appropriate studies. Based on the characteristics of our study, we felt it could afford more reliable and accurate information.

We attributed the result that the LR group was significantly higher than the TA group in terms of 5-year OS and 1-year DFS in our research to the following factors: First, the majority of patients we included were very early-stage HCC, having a well-preserved liver function and sufficient residual liver volume and being suitable for LR to complete eradication of the tumor. Therefore, the LR group got better long-term survival compared to the TA group [25, 26]. Secondly, the tumor size included in our study was relatively small, making it easy for surgeons to get the surgical margins. Third, MWA is an alternative procedure that equals RFA. Though the previous meta-analyze showed patients with MWA got higher 3-year and 5-year OS than the LR group [27], our meta-analysis included study of MWA treatment versus LR therapy. Thus, the TA group cannot get benefit from MWA treatment. Finally, Santambrogio et al. [18] showed that local tumor progression and intra-segmental recurrence were significantly higher in the ablation therapies group. An insufficient primary tumor control ability was likely responsible for early local recurrences. By reducing local recurrences, LR was more likely to be effective than RFA when it comes to complete treatment.

Several limitations existed in this study. First, despite using the scientific PSM method to get rational matching of two groups, there was still inter-group heterogeneity, such as the selection for different RFA electrodes, the tumor location, and the method of isolation for RFA. Therefore, we need prospective multicenter RCTs to compare the therapeutic efficacy of the TA and LR for HCC patients to reduce inter-group heterogeneity. Furthermore, in two trials out of five, the follow-up period did not exceed five years. Thus long-term survival rates based on pooled ORs might provide less proof than short-term survival rates. Third, the underlying causes of liver disease differing between countries (Eastern vs. Western) might have affected outcomes.

## Conclusion

In conclusion, liver resection offered better long-term survival and disease-free survival than thermal ablation for cirrhotic patients with HCC based on our meta-analysis.

## Supplementary Information

Below is the link to the electronic supplementary material.

Supplementary file1 (DOCX 77 KB)
