# Supplementary material for: Thermal ablation versus liver resection for hepatocellular carcinoma in patients with cirrhosis: a systematic review and meta-analysis of propensity-score matched studies
Source: Clin Exp Med. 2024 Feb 1;24(1):32. doi: 10.1007/s10238-023-01285-w (PMC10834626; doi:10.1007/s10238-023-01285-w)
Supplement: Supplementary file 1 — Supplementary file1 (DOCX 77 KB) [file 10238_2023_1285_MOESM1_ESM.docx]

Supplementary Files

a


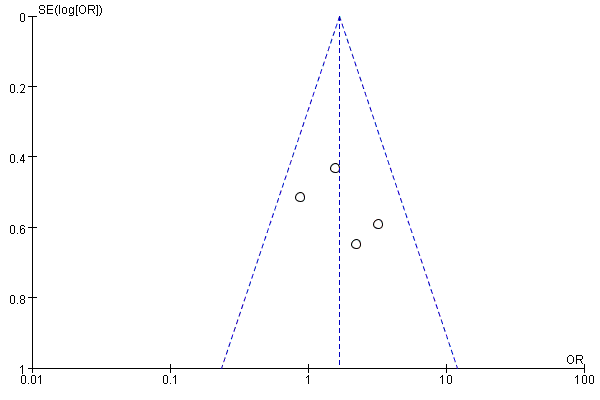


b


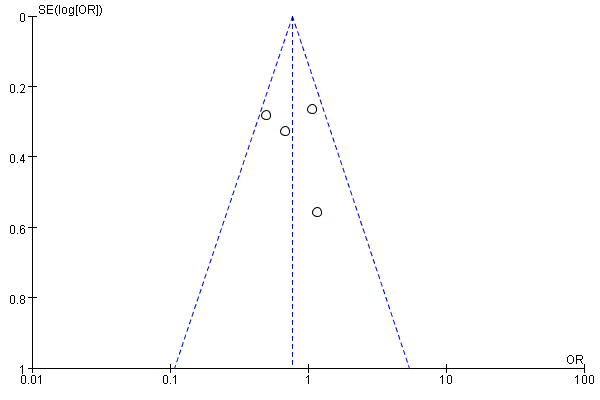


c


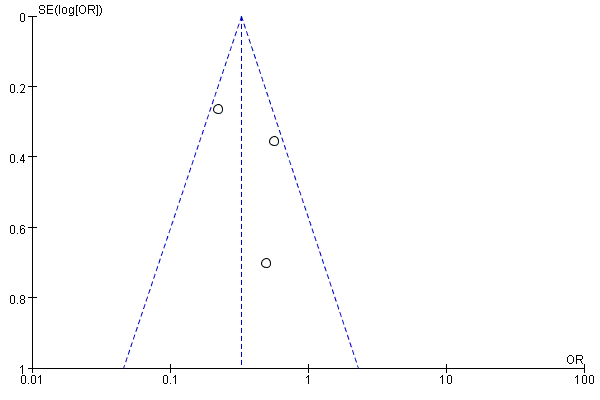


Fig. S1. Funnel plots to detect the publication bias for the (a) 1-year OS, (b) 3-year OS, and (c) 5-year OS.

a


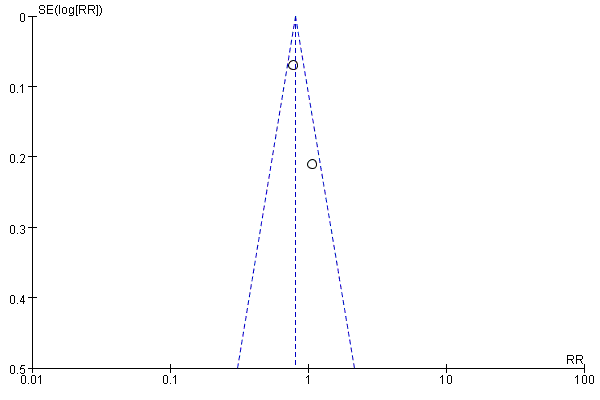


b


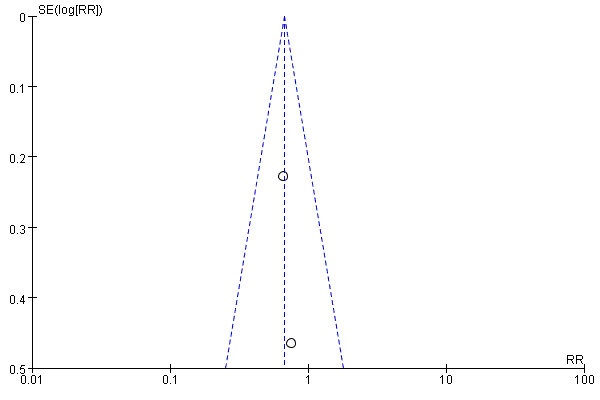


c


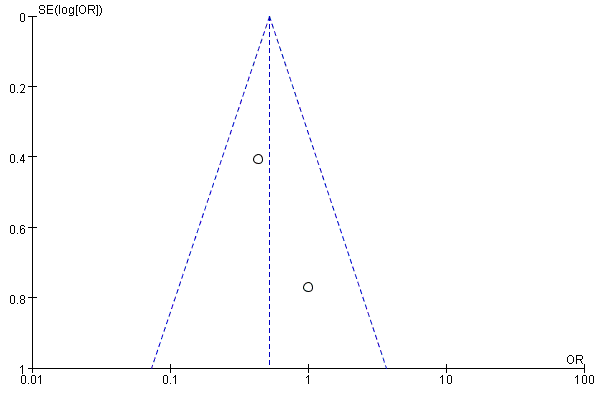


Fig. S2. Funnel plots to detect the publication bias for the (a) 1-year DFS, (b) 3-year DFS, and (c) 5-year DFS.

a


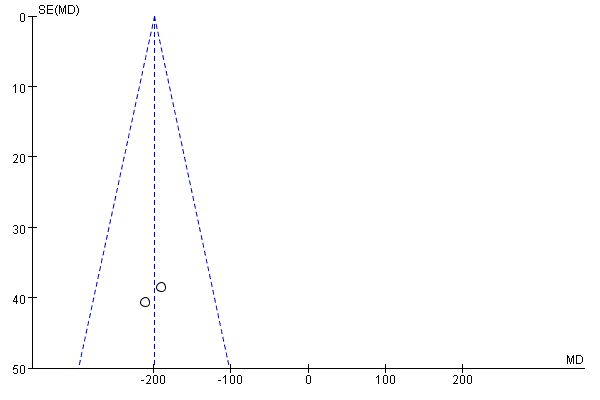


b


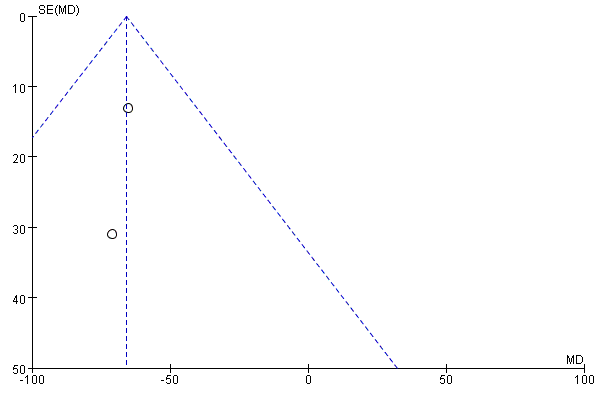


c


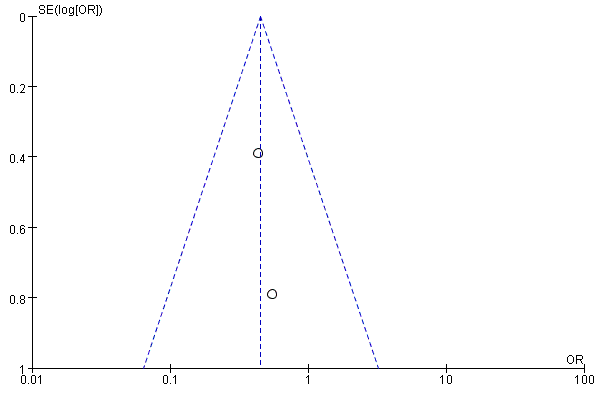


d


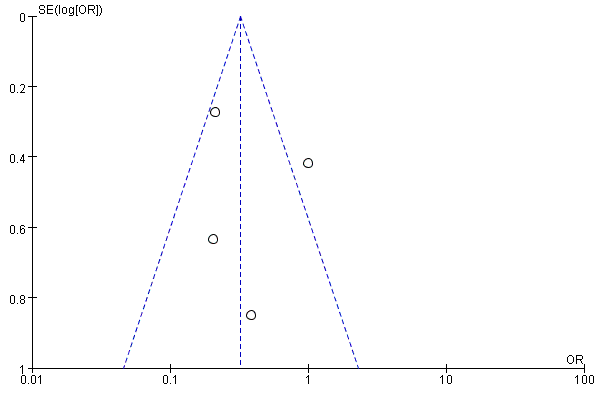


Fig. S3. Funnel plots to detect the publication bias for the (a) operative time, (b) hospital stay, (c) perioperative blood transfusion rate, and (d) major complications rate.
